# Quantitative peritumoral magnetic resonance imaging fingerprinting improves machine learning-based prediction of overall survival in colorectal cancer

**DOI:** 10.37349/etat.2024.00205

**Published:** 2024-02-19

**Authors:** Azadeh Tabari, Brian D’Amore, Janice Noh, Michael S. Gee, Dania Daye

**Affiliations:** University of Campania “L. Vanvitelli”, Italy; ^1^Department of Radiology, Massachusetts General Hospital, Boston, MA 02114, USA; ^2^Harvard Medical School, Boston, MA 02115, USA; ^3^Department of informatics, Boston University, Boston, MA 02114, USA

**Keywords:** Colorectal cancer, peritumoral texture features, imaging biomarkers, survival

## Abstract

**Aim:**

To investigate magnetic resonance imaging (MRI)-based peritumoral texture features as prognostic indicators of survival in patients with colorectal liver metastasis (CRLM).

**Methods:**

From 2007–2015, forty-eight patients who underwent MRI within 3 months prior to initiating treatment for CRLM were identified. Clinicobiological prognostic variables were obtained from electronic medical records. Ninety-four metastatic hepatic lesions were identified on T1-weighted post-contrast images and volumetrically segmented. A total of 112 radiomic features (shape, first-order, texture) were derived from a 10 mm region surrounding each segmented tumor. A random forest model was applied, and performance was tested by receiver operating characteristic (ROC). Kaplan-Meier analysis was utilized to generate the survival curves.

**Results:**

Forty-eight patients (male:female = 23:25, age 55.3 years ± 18 years) were included in the study. The median lesion size was 25.73 mm (range 8.5–103.8 mm). Microsatellite instability was low in 40.4% (38/94) of tumors, with Ki-ras2 Kirsten rat sarcoma viral oncogene homolog (*KRAS*) mutation detected in 68 out of 94 (72%) tumors. The mean survival was 35 months ± 21 months, and local disease progression was observed in 35.5% of patients. Univariate regression analysis identified 42 texture features [8 first order, 5 gray level dependence matrix (GLDM), 5 gray level run time length matrix (GLRLM), 5 gray level size zone matrix (GLSZM), 2 neighboring gray tone difference matrix (NGTDM), and 17 gray level co-occurrence matrix (GLCM)] independently associated with metastatic disease progression (*P* < 0.03). The random forest model achieved an area under the curve (AUC) of 0.88.

**Conclusions:**

MRI-based peritumoral heterogeneity features may serve as predictive biomarkers for metastatic disease progression and patient survival in CRLM.

## Introduction

Liver metastasis develops in approximately 50% of patients with colorectal cancer (CRC) during their disease [1]. Hepatic resection is the most effective treatment option for colorectal liver metastasis (CRLM) with a reported 40% to 60% 5-year survival [2, 3]. Patients with potentially resectable CRLM usually undergo neoadjuvant chemotherapy to achieve shrinkage of the liver tumors [2, 4]. Response assessment after chemotherapy mainly consists of visually evaluating changes in lesion size and morphology [5]. The most commonly used system to assess the response to treatment in clinical trials and daily practice is response evaluation criteria in solid tumors (RECIST) [6]. However, a limitation of RECIST is that size measurements fail to capture microstructural changes related to intralesional necrotic changes, which are known to be significant factors correlated with treatment response [7]. In cases where necrotic changes occur in metastatic lesions without a drastic size reduction due to successful treatment, RECIST will fail to recognize a treatment response. Moreover, chemotherapy can impair the assessment of lesions by affecting the liver parenchyma [8, 9]. Identifying early predictors for survival after chemotherapy is crucial because it would help personalize follow-up, propose precocious complementary procedures, and create opportunities for neoadjuvant treatment optimization based on the anticipated treatment response. Recently, radiomics investigation of the area immediately surrounding the tumor mass, peritumoral region, has become a topic of interest among radiologists. The rationale is that the tumor microenvironment might harbor valuable disease-specific prognostic cues [10, 11]. Several studies have shown the potential of radiomics of the peritumoral region in predicting clinical outcomes in breast, lung, and esophageal cancer [10, 12–14]. However, the significance of peritumoral radiomics in patients with CRLM has not been established yet. The goal of this study was to investigate the role of peritumoral heterogeneity in predicting overall survival (OS) in patients with metastatic CRC.

## Materials and methods

### Patients

In this institutional review board (IRB) approved retrospective study, forty-eight patients with biopsy proven stage IV CRC with liver metastasis who underwent treatment with front-line standard chemotherapy protocols [including folinic acid, fluorouracil, and oxaliplatin (FOLFOX) and fluorouracil, folinic acid, and irinotecan (FOLFIRI)] and/or radiotherapy were identified, between January 2007 and December 2015. All patients met the following criteria: (a) biopsy proven colorectal adenocarcinoma; (b) presence of CRLM; (c) T1-weighted post-contrast magnetic resonance imaging (MRI) within 3 months prior to preoperative FOLFIRI or FOLFOX-based chemotherapy. The treatment of all patients was carried out by a team of oncologists at the Massachusetts General Hospital Cancer Center to ensure the availability of follow-up data. Clinical responders were defined as patients who experienced a partial or complete response according to RECIST or patients who had stable disease.

### MRI protocol

A contrast-enhanced abdominal MRI was acquired within 3 months before starting treatment. All the liver MRI exams were done on either a 1.5T MRI scanner (*n* = 40, Signa HDxt GE Medical Systems and Magnetom Avanto, Siemens Healthcare) or a 3T magnet (*n* = 8, Discovery 750MR GE Medical Systems and Magnetom Trio, Siemens Healthcare) with a body coil positioned over the abdomen. Contrast enhanced T1-weighted sequences were obtained and, for this study, the portal venous phase (60–70 s post-contrast) images were selected. Intravenous gadolinium contrast (gadopentetate, Magnevist, Bayer; gadoterate, Dotarem, Guerbet; or gadoxetate, Eovist, Bayer) was administered at the standard approved clinical dose (0.1 mmol/kg for gadopentetate and gadoterate, 0.025 mmol/kg for gadoxetate) by power injector at a rate of 1 mL/s [15].

### Data collection

Patient age, sex, lesion size, location of primary tumor, tumor Ki-ras2 Kirsten rat sarcoma viral oncogene homolog (*KRAS*) status, tumor microsatellite instability (MSI), stage of colon cancer, radiotherapy status, chemotherapy regimen, and follow-up duration were collected from the medical records. All-cause mortality was the assessed outcome.

### Image segmentation and radiomics feature extraction

The Digital Imaging and Communications in Medicine (DICOM) images were reviewed by radiologists with expertise in liver imaging and blinded to the clinical findings to identify the CRLM. The metastases were semi-automatically segmented by an instructor in radiology (with > 7 years of experience in tumor segmentation) on portal venous phase T1-weighted images using three-dimensional (3D) slicer (version 4.8.1) [15]. The window levels were adjusted to optimize visualization of the liver mass. Manual delineations included a 10 mm region surrounding each segmented tumor and semi-automatic volumetric segmentation methods were applied using 3D slicer. A multiparametric vector was derived from each lesion. All images were normalized prior to extracting quantitative features.

**Figure 1:**
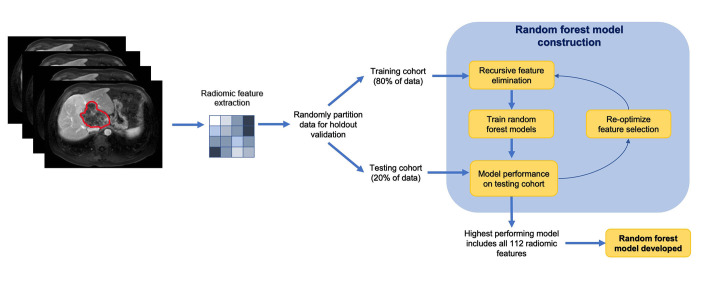
Radiomics workflow and analysis in a stepwise approach *Note.* Adapted from “Machine learning-based radiomic features on pre-ablation MRI as predictors of pathologic response in patients with hepatocellular carcinoma who underwent hepatic transplant,” by Tabari A, D’Amore B, Cox M, Brito S, Gee MS, Wehrenberg-Klee E, et al. Cancers. 2023;15:2058 (https://doi.org/10.3390/cancers15072058). CC BY.

A random forest-based machine learning model was applied to predict the outcome (i.e., patient survival). The segmented images and masks of patients were first normalized and resampled. The tumor mask file denotes the tumor region with 1’s. The peritumoral mask of different sizes was obtained from the initial tumor mask by changing pixels of distance “n” or less from each of the pixels noted as tumor as 1’s (where “n” changed depending on the size). Then, the pixels noted as tumors were changed to 0’s, such that when analyzing, the peritumoral data would exclude the tumor region. A vector consisting of 112 radiomic features (shape, first-order, texture) was derived from 94 peritumoral masks across a total of 48 patients using the Pyradiomics package in Python (version 4.2). The features consisted of first-order features, two-dimensional (2D) and 3D shape features, gray level co-occurrence matrix (GLCM) features, gray level dependence matrix (GLDM) features, gray level size zone matrix (GLSZM) features, gray level run time length matrix (GLRLM) features, and neighboring gray tone difference matrix (NGTDM) features. The dataset was partitioned into training and testing sets at a ratio of 4:1. Recursive feature elimination was applied to select and test the effectiveness of different numbers of radiomic features. Ultimately, however, using all features produced the best results. The area under the curve (AUC) for using radiomic features using the random forest algorithm was generated by receiver operating characteristic (ROC) analysis. The radiomics workflow and the development of machine learning model is demonstrated in Figure 1.

### Statistical analysis

The survival prediction score was derived from the Cox model with Kaplan-Meier survival curve, for each patient. The contribution of each texture feature to OS prediction was assessed using univariate logistic regression analysis. *P* < 0.05 was considered statistically significant on the Wald test. All statistical analyses and machine learning models were performed using Python 4.2 (https://www.python.org/).

## Results

### Patient characteristics

Forty-eight patients (25 females, age 55.3 years ± 18 years) corresponded with 94 metastatic tumors were included. The mean lesion size was 32 mm ± 19 mm (range 85–103.8 mm). Twenty-nine out of forty-eight (60.4%) patients were *KRAS*-positive. Seventeen out of forty-eight (35.4%) patients had low MSI. Primary tumor sites were left and sigmoid colon (35/48, 72.9%), right colon (11/48, 22.9%), and transverse colon (2/48, 4.2%). Metastatic disease was limited to the liver in 52.1% (25/48) of the patients. Forty-seven out of forty-eight (97.9%) of the study population were treated with chemotherapy and 35.4% (17/48) received chemoradiotherapy for their liver metastases. Seventeen out of forty-eight (35.4%) patients exhibited local tumor progression in 28/48 (58.3%) lesions. The clinicobiological characteristics of the patients and liver lesions are encapsulated in Table 1.

**Table 1:** Patient and liver tumor characteristics prior to the start of treatment

**Clinical variables**	**Value**
Number of subjects	48
Age^a^ [mean ± standard deviation (SD), years]	55.3 ± 18
Sex (female:male)	25:23
Number of lesions	94
*KRAS* positive	29/48 (60.4%)
Extrahepatic disease	23/48 (47.9%)
The median time between MRI and chemotherapy (days)	23
Local tumor progression	17/48 (35.4%)
Chemoradiotherapy	17/48 (35.4%)
Follow-up (months)	34.9 21
Location of primary tumor	
Ascending colon	35/48 (72.9%)
Transverse colon	2/48 (4.2%)
Descending and sigmoid colon	11/48 (22.9%)
MSI	
No testing available	30/48 (62.5%)
MSI-low (L)	17/48 (35.4%)
MSI-high (H)	1/48 (2.1%)
Tumor number	
1	31/48 (64.6%)
> 1	17/48 (35.4%)
TNM classification	
II or III	4/48 (8.3%)
IV	44/48 (91.7%)
Tumor size	
≤ 3 cm	61/94 (64.9%)
> 3 cm	33/94 (35.1%)
Chemotherapy regimen	
FOLFIRI	4/48 (8.3%)
FOLFOX	44/48 (91.7%)

^a^ at the time of MRI

### Survival analysis

**Figure 2:**
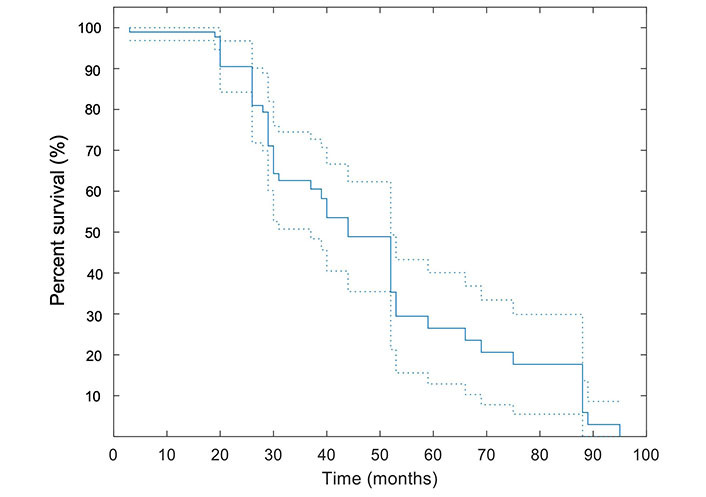
Kaplan-Meier curve for OS. A pointwise 95% band is shown (dashed lines). All patients were included in this analysis

The median OS for the study population was 29 [95% confidence interval (CI): 25–35] months. In this study, the mean follow-up duration was 34.9 months ± 21.6 months. Twenty-one out of forty-eight (43.8%) patients were deceased at the end of the study including: 52.9% (9/17) of patients with local tumor progression and 38.7% (12/31) of patients without local progression (*P* = 0.56). The median OS of 17 patients with local tumor progression and 31 patients who showed no local tumor progression were 25.5 months and 30 months, respectively; the difference was not statistically significant. The survival data was available for up to 95 months. The Kaplan-Meier curve illustrating the survival estimates of the study population is included in Figure 2.

### MRI-based radiomics features

A total of 112 radiomic features were derived from a 10 mm region surrounding each segmented tumor. Univariate logistic regression and Wald test results (Table 2) showed 42 features including: 8 first order, 5 GLDM, 5 GLRLM, 5 GLSZM, 2 NGTDM, and 17 GLCM to be independently correlated with the prediction of patients’ OS (all *P* < 0.05 on Wald test).

**Table 2:** Statistically significant radiomic features on univariate logistic regression and Wald test (*P* < 0.05)

**Parameters**	**Features**	**Coeff (95% CI)**	***P* < 0.05**
First order	10th Percentile	0.78 (1.34, 0.19)	0.01
	90th Percentile	0.80 (1.4, 0.2)	0.01
	Entropy	–1.82 (–0.22, –3.42)	0.03
	Maximum	0.45 (0.87, 0.03)	0.04
	Mean	0.95 (1.61, 0.3)	0.01
	Median	0.88 (1.50, 0.26)	0.01
	Root mean squared	1.10 (1.82, 0.38)	0.01
	Uniformity	3.86 (7.37, 0.35)	0.03
GLCM	Cluster prominence	–1.45 (–0.22, –2.69)	0.02
	Cluster tendency	–2.06 (–0.09, –4.03)	0.04
	Contrast	–24.1 (–5.68, –42.5)	0.01
	Difference average	–24.1 (–5.68, –42.5)	0.01
	Difference entropy	–4.62 (–1.19, –8.06)	0.01
	Difference variance	–26.2 (–6.40, –45.9)	0.01
	Inverse difference (Id)	48.1 (84.9, 11.4)	0.01
	Inverse difference moment (Idm)	48.1 (84.9, 11.4)	0.01
	Inverse difference moment normalized (Idmn)	120.3 (212.3, 28.4)	0.01
	Inverse difference normalized (Idn)	72.2 (127.4, 17)	0.01
	Informational measure of correlation 2 (Imc2)	–1.92 (–0.27, –3.56)	0.02
	Inverse variance	–24.1 (–5.68, –42.5)	0.01
	Joint energy	3.54 (6.57, 0.50)	0.02
	Joint entropy	–1.42 (–0.27, –2.57)	0.02
	Maximum probability	4.54 (8.86, 0.22)	0.04
	Sum entropy	–1.50 (–0.27, –2.73)	0.02
	Sum squares	–7.86 (–0.73, –15)	0.03
GLDM	Dep variance	–0.19 (–0.03, –0.35)	0.02
	Gray level variance	–7.71 (–0.7, –14.7)	0.03
	Large dep emphasis	0.03 (0.06, 0.01)	0.01
	Small dep emphasis	–1,509.4 (–468.9, –2,549.9)	0.01
	Small dep high gray level emphasis	–107.8 (–5.81, –209.7)	0.04
GLRLM	Gray level non-uniform normalized	3.36 (6.29, 0.44)	0.02
	Gray level variance	–6.73 (–0.88, –12.6)	0.02
	Run percentage	–32.3 (–9.86, –54.8)	0.01
	Short run emphasis	–13.1 (–3.98, –22.3)	0.01
	Short run high gray level emphasis	–2.3 (–0.52, –4.07)	0.01
GLSZM	Small area emphasis	–4.88 (–1.23, –8.48)	0.01
	Small area high gray level emphasis	–4.15 (–1.21, –7.09)	0.01
	Small area low gray level emphasis	–4.33 (–0.49, –8.18)	0.03
	Zone entropy	–0.30 (–0.01, –0.59)	0.05
	Zone percentage	–1,362.4 (–174.1, –2,550.6)	0.03
NGTDM	Complexity	–26.1 (–6.31, –45.9)	0.01
	Contrast	–141.7 (–14.3, –269)	0.03

**Figure 3:**
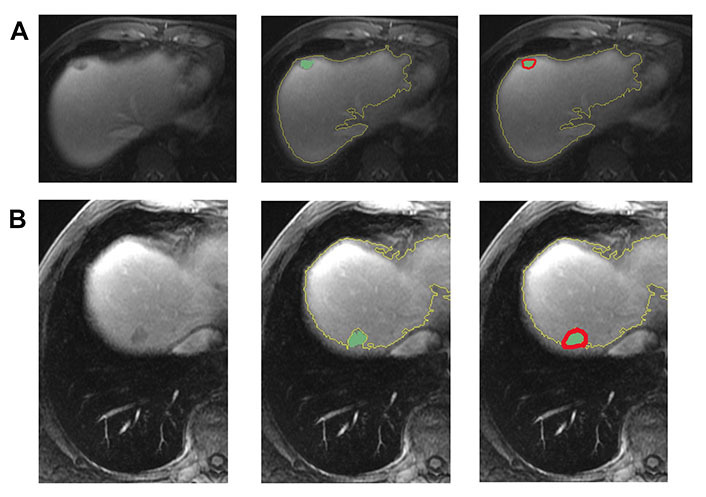
Representative images demonstrating step by step semiautomatic lesion delineation in 2 patients with different survival outcomes. (A) A 47-year-old male with a 1.5 cm *KRAS*-positive, MSI-L hepatic metastasis. This patient was alive 45 months following imaging; (B) a 51-year-old male with a 1.8 cm neuroblastoma rat sarcoma (RAS) viral oncogene homolog (*NRAS*)-positive, MSI-L liver metastasis. This patient died at 11 months following imaging

In Figure 3, two representative MRI images illustrate tumor heterogeneity for a patient who survived 45 months and another who died at 11 months follow-up. The metastatic tumor was comparable in size for both patients with transverse colon as the primary site of disease.

### Machine learning for survival prediction

**Figure 4:**
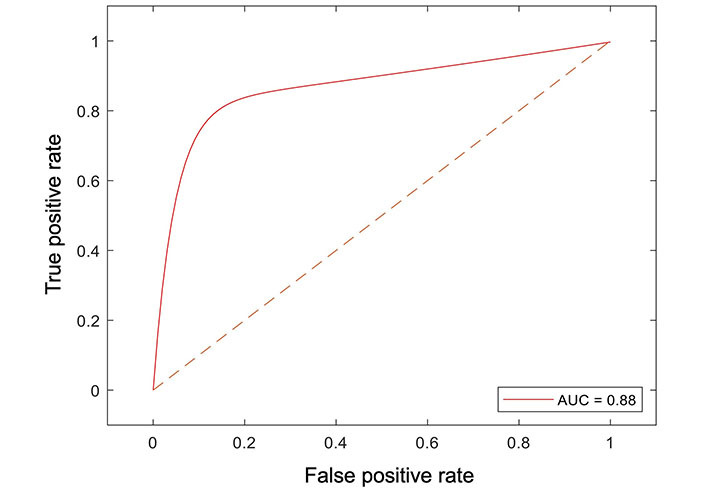
Receiver operator curve analysis of the machine learning model for peritumoral features and survival

All radiomic features were included in a random forest-based machine learning model to predict OS. The dataset was partitioned at a ratio of 4:1 for the training and testing set. The final model resulted in an AUC of 0.88 in the testing dataset (Figure 4).

## Discussion

This study represents the first attempt to ascertain the value of peritumoral region radiomics features [extracted from pre-treatment magnetic resonance (MR) images] in predicting OS in patients with CRLM. Radiomics, a promising non-invasive tool, holds the potential to forecast prognosis and enhance treatment optimization in the day-to-day care of cancer patients. Previous studies have reported individual radiomic features as predictive markers for response to treatment, disease recurrence, and OS [16, 17]. While the primary focus of most radiomics studies has centered on the intra-tumoral region, peritumoral enhancement patterns have demonstrated efficacy as indicators of microinvasion, tumor biological aggressiveness, and micrometastasis [18–21]. In this study, the hypothesis was that capturing peritumoral expansion (10 mm) through radiomics could enhance the performance of OS assessment before starting the treatment. In previous publications, peritumoral information was incorporated by extracting features from a region of interest (ROI) around the tumor, while in this study it was precisely defined a 10 mm peritumoral margin and features were derived from this specific ROI [16, 17]. Feature extraction was carried out automatically, eliminating the need for visual assessment by radiologists.

In the current study, 42 out of 112 radiomics features derived from the pretreatment MRI were found to be associated with the patient outcomes and were utilized for constructing the radiomics signature. These features encompassed six categories: first order, GLDM, GLRLM, GLSZM, NGTDM, and GLCM. This reflected that different-order texture heterogeneity could quantitatively evaluate peritumoral region heterogeneity, exerted strong predictive power for clinical outcomes, and played a crucial role in the construction of the radiomics signature.

The results exhibit promises in predicting treatment outcomes using radiomic features derived from the peritumoral region on pre-treatment MRI exams, achieving an AUC of 0.88. Prior studies have indicated that a machine learning-based radiomics analysis of pre-ablation computed tomography (CT) could serve as an imaging biomarker to predict local tumor progression with curative intent for CRLM patients [21]. In patients with hepatocellular carcinoma (HCC), radiomics information from tumor margin has shown an association with microvascular invasion, albeit with a slightly lower AUC compared to tumor radiomics alone. Notably, the performance of the model declined when combining intra- and peritumoral radiomics features, suggesting that information from the peritumoral region may not complement information from the tumor [17, 18, 22]. However, in studies done by Shan et al. [16] and Kim et al. [23], the peritumoral radiomics model achieved superior performance to the intra-tumoral radiomics model (AUC 0.79 *vs.* 0.62 and AUC 0.71 *vs.* 0.69, respectively) for evaluating early recurrence of HCC after curative tumor resection or ablation.

This study possesses several strengths. Firstly, the cohort comprised both large and smaller tumor sizes with 64.9% having a size of ≤ 3 cm and 35.1% > 3 cm. Secondly, tumor ROIs were drawn semi-automatically, ensuring consistency in peritumoral region size and avoiding potential mistakes associated with manually drawing lesion boundaries, which might be susceptible to subjectivity. This could be a factor that positively influenced the accuracy of the model. Thirdly, radiomics features were extracted from the peritumoral 3D region (volume) rather than through a 2D image slice segmentation. Volumetric segmentation and analysis offer improved lesion assessments and result in fewer sampling errors compared to single slice segmentation. Fourthly, despite the use of MRI data with different field strengths in this study, the radiomics results remained unaffected. This is because intensity standardization was applied to all images before computing the radiomics features, addressing any inter-subject intensity variations.

**Figure 5:**
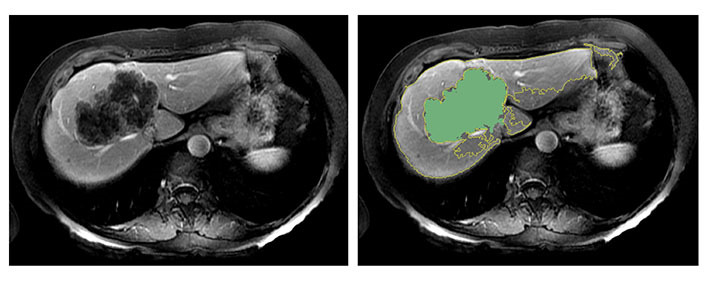
The semiautomatic delineation identified the tumor of 97 mm in diameter without including the extrahepatic region

It is important to note that in this study, the extracted peritumoral region may include extrahepatic areas. This is partly due to the relatively large size of some tumors in the cohort. As illustrated in Figure 5, there were cases where volumetric segmentation of the large tumor margin did not encompass extrahepatic regions. The presence of extrahepatic areas may contribute to the heterogeneity in marginal radiomics feature extraction. This observation may be linked to the chosen margin size (i.e., 10-pixel).

Selecting a wider margin would lead to the inclusion of larger portions of extra-hepatic tissue potentially worsening the heterogeneity issue, while a narrower margin may not provide sufficient information. In this study, a 10-pixel (10 mm wide) margin was chosen, aligning with choices made in previous publications [24, 25]. However, further investigation is required to establish the ideal margin size.

This study is constrained by its retrospective single-center design and a relatively small patient cohort. Inevitably, selection bias may exist, potentially influencing the analysis, and several clinical variables could not be measured. Additionally, there were variations in the treatment regimen used (chemotherapy *vs*. chemoradiotherapy). Parameter optimization was performed using 3-fold cross-validation to create a generalized model and minimize the risk of overfitting. To ensure the generalizability of our findings, future work should involve an external validation study across multiple centers.

In conclusion, machine learning-based predictive models that incorporate radiomic features from the peritumoral region may enhance the identification of lesions with better OS prior to initiating treatment. This information could be valuable for treatment optimization in patients with CRLM. In the next phase of this study, the goal is to conduct an external validation in a multicenter setting.

## Abbreviations

3D: three-dimensional
AUC: area under the curve
CRC: colorectal cancer
CRLM: colorectal liver metastasis
FOLFIRI: fluorouracil, folinic acid, and irinotecan
FOLFOX: folinic acid, fluorouracil, and oxaliplatin
GLCM: gray level co-occurrence matrix
GLDM: gray level dependence matrix
GLRLM: gray level run time length matrix
GLSZM: gray level size zone matrix
MRI: magnetic resonance imaging
MSI: microsatellite instability
NGTDM: neighboring gray tone difference matrix
OS: overall survival
RECIST: response evaluation criteria in solid tumors
ROI: region of interest

## Declarations

### Availability of data and materials

The datasets for this manuscript are not publicly available because data contains personally identifiable information that may be restricted from public access to protect individuals’ privacy. Requests for accessing the datasets should be directed to (Azadeh Tabari, atabari@mgh.harvard.edu).

### Copyright

© The Author(s) 2024.
